# Scleractinian corals incorporate microplastic particles: identification from a laboratory study

**DOI:** 10.1007/s11356-021-13240-x

**Published:** 2021-03-15

**Authors:** Florian Hierl, Henry C. Wu, Hildegard Westphal

**Affiliations:** 1 Leibniz Centre for Tropical Marine Research (ZMT), Fahrenheitstraße 6, 28359 Bremen, Germany; 2 Faculty of Geosciences, University of Bremen, Klagenfurter Straße 4, 28359 Bremen, Germany

**Keywords:** Incorporation, Encrustation, Spectroscopy, Microplastic, Calcification, Coral skeleton

## Abstract

Microplastics have been detected on beaches and in the ocean from surface habitats to the deep-sea. Microplastics can be mistaken for food items by marine organisms, posing a potential risk for bioaccumulation and biomagnification in the food chain. Our understanding of microplastic pollution effects on ecosystem and physiological processes of coral reefs is still limited. This study contributes to the understanding of effects of microplastic pollution on skeletal precipitation of hermatypic corals. In a five month aquarium-based experiment, specimens of four tropical species were temporarily exposed to high concentrations (ca. 0.5 g L^-1^) of polyethylene terephthalate (PET) microplastic particles (< 500 μm). The coral specimens all survived this treatment and show skeletal growth. The skeletal material produced during the experiment, however, incorporated plastic particles and plastic fibres in the aragonitic structure. Long-term consequences of such inclusions on skeletal properties such as stability are yet unknown.

**Supplementary Information:**

The online version contains supplementary material available at 10.1007/s11356-021-13240-x.

## Introduction

In the past five decades, marine environments have been exposed to increasing amounts of plastic pollution, recently gaining considerable attention in the scientific community (Stefatos et al. 1999; GESAMP 2015). Pollution by plastic litter in the oceans was first reported in the early 1970s (Carpenter and Smith 1972; Carpenter et al. 1972; Fowler 1987), but only recognized later as an urgent topic (Stefatos et al. 1999; Andrady 2011). The amount of synthetic plastic pieces entering the marine ecosystem is alarming, and has been estimated to sum up to some 12.7 million tons per year (Jambeck et al. 2015).

All marine environments are thought to be subject to plastic pollution, ranging from shallow surface waters to deep-sea habitats (Fischer et al. 2015). The widespread dispersion of many types of plastics across all marine ecosystems derives from their buoyancy in seawater (Derraik 2002; Reisser et al. 2015). The highest plastic concentrations of floating pieces can be found accumulated in the oceanic gyres (Berloff et al. 2002; Maximenko et al. 2012; Eriksen et al. 2014).

Recent studies show that besides the large amounts of macroplastics floating on the sea surface, an unknown portion of microplastic particles (generally defined by a size <5 mm) pollutes the marine realm (Moore 2008; Andrady 2011; Wright et al. 2013; Eriksen et al. 2013; GESAMP 2015). These particles occur in a wide variety of shapes and sizes throughout the water column and are being deposited in sediments around the globe (Fischer et al. 2015; Van Cauwenberghe et al. 2015; Kane et al. 2020).

Marine plastic litter mostly derives from package materials from land-based sources (Pruter 1987; Gregory 1991; Derraik 2002). Polyethylene (PE), polyethylene terephthalate (PET), polystyrene (PS), polypropylene (PP), and polyvinyl chloride (PVC) are the most common types of plastic in the marine environment (Andrady 2011). Two types of marine microplastics can be distinguished (Cole et al. 2011). Particles that already have the shape and size of microplastics when entering the marine environment are called primary microplastics and are produced for cosmetics, hand and facial cleaners, or are by-products of air-blasting and sewage plants (Fendall and Sewell 2009; Browne et al. 2010; Cole et al. 2011). Secondary microplastics are derived from the processes of degradation of larger plastic pieces and make up the larger portion of the two (Barnes et al. 2009; Wang et al. 2016). Decomposition and therefore the creation of secondary microplastics are driven by thermal-oxidative, photolytic, abrasive, and biotic processes (Andrady 2011; Gewert et al. 2015; Weinstein et al. 2016).

Due to their size and the usage of toxic components during plastic production, microplastics are considered to be a threat to marine organisms, including scleractinian corals (Hall et al. 2015; Hahladakis et al. 2018; Reichert et al. 2018, 2019; Hankins et al. 2018). Increasing numbers of studies report potential risks of microplastic pollution on coral health. The results are based on laboratory studies (Hall et al. 2015; Allen et al. 2017; Reichert et al. 2018, 2019; Martin et al. 2019) and environmental assessments (Connors 2017; Rotjan et al. 2019; Ding et al. 2019; Tan et al. 2020).

Laboratory studies have demonstrated that corals ingest and egest microplastic particles (Hall et al. 2015; Allen et al. 2017; Reichert et al. 2018; Hankins et al. 2018). Elevated concentrations of ingested microplastics in the mesenterial gut cavity tissue are expected to be a risk for coral health (Hall et al. 2015) because plastic fragments are not broken down in the gut (Allen et al. 2017), potentially resulting in gut blockage (Stamper et al. 2006), false satiation and reduced energy resources (Wright et al. 2013; Watts et al. 2015). Corals have been shown to ingest microplastic particles almost ten times more frequently than sediment particles of comparable size, the preference for plastics been caused by phagostimulants contained in plastic (Allen et al. 2017).

Additionally, direct contact with microplastic particles can cause tissue necrosis, overgrowth, and even migration into the skeleton-tissue interface (Reichert et al. 2018; Ding et al. 2019). The effects of microplastic particles on coral health and coral growth rate appear to be species-specific and also depend on the duration of exposure to microplastics (Hankins et al. 2018; Reichert et al. 2019).

Another dimension of microplastic impact on coral health emerges from contaminated microplastic particles e.g. with polycyclic aromatic hydrocarbons (PAHs), persistent organic pollutants (POPs), heavy metals or pathogens resulting in increased spreading of diseases amongst coral colonies (Rios et al. 2007; Teuten et al. 2007, 2009; Ashton et al. 2010; Bakir et al. 2012; Fisner et al. 2013; Kirstein et al. 2016; Lamb et al. 2018; Hahladakis et al. 2018; Rotjan et al. 2019). Several studies tested the adhesion potential, as well as the plastic properties that lead to ingestion and egestion, the amount ingested over a given time, and the physiological effects of microplastics on growth rates in scleractinian corals (Hall et al. 2015; Allen et al. 2017; Reichert et al. 2018, 2019; Hankins et al. 2018; Martin et al. 2019; Corona et al. 2020). The processes following ingestion and overgrowth, however, are still poorly understood.

The present study investigates the impacts of microplastics on the precipitation of skeletal material of reef-building scleractinian corals to gain knowledge on long-term exposure risks in polluted waters. Special focus is on encrustations of plastic particles and fibres in coral skeletal material. The mesocosm tank experiment reported here extended over a time interval of five months and involved four different scleractinian coral species. Four coral species were chosen, namely, *Acropora valida* [Dana, 1846], *Montipora capricornis* [Veron, 1985], *Pocillopora damicornis* [Linnaeus, 1758] and *Seriatopora hystrix* [Dana, 1846] that represent different morphologies. The aim was to test whether the shape of the coral influences the encrustation potential of microplastic in the skeleton, analogous to the influence of morphology on the effect of carbonate and siliciclastic sediment grains, where a branching morphology is favourable in terms of sediment rejection compared to a plate-like morphology (Duckworth et al. 2017).

## Material and methods

### Aquaria setup

Specimens of the four coral species (*A. valida*, *M. capricornis*, *P. damicornis* and *S. hystrix*) were exposed to microplastic particles for 24 hours every two weeks over five months in a fully controlled lab experiment. These four hermatypic species were chosen because of their structural and morphological differences and differences in polyp sizes. *S. hystrix*, *P. damicornis* and *A. valida* are branching corals, while *M. capricornis* has a plate-like growth. *P. damicornis* has the largest polyp cavity compared to the other species. In *S. hystrix* the polyps are positioned much closer to each other compared to *P. damicornis* and *A. valida*.

Before the experiment, coral clones were produced by taking coral fragments of 4–10 cm length from a parent colony cultured at the Leibniz Centre for Tropical Marine Research (ZMT) aquaria facility in Bremen, Germany. These were glued onto ceramic stubs using a one-component glue (Coral Glue, EcoTech Marine, Allentown, PA, USA) and placed in mesocosm tanks at this aquarium facility which utilizes a fully recirculating system with artificial seawater using Red Sea salts. The microbiome in the tanks was allowed to stabilise for an 8-week phase before the corals were introduced in. The coral fragments were then acclimatised for six weeks prior to the start of the experiment.

**Fig. 1:**
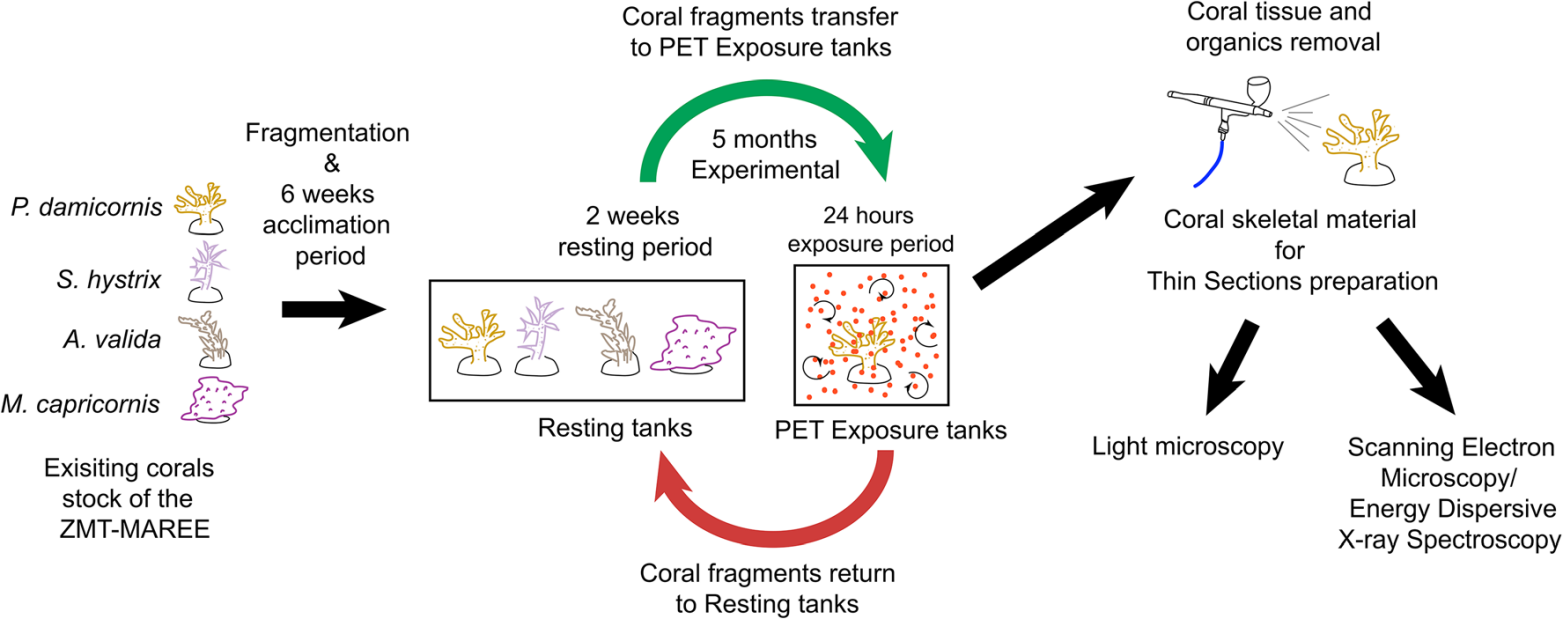
Schematic diagram of the experimental setup and exposure cycles. The diagram shows the fragmentation, the transfer of fragments between microplastic exposure and resting tanks and the post-experimental processing steps

The experimental design included four hosting tanks and four separate exposure tanks. Corals were held in the hosting tanks during breaks between exposure cycles. Of the four exposure tanks, two were equipped with a constant circulation of microplastics, while two tanks for control treatment did not contain microplastics (Fig. 1). Each of the four hosting tanks held 16 coral fragments (i.e. four per species), summing up to 64 fragments for the experiment. Corals were fed with live *Artemia* (10 ml *Artemia*-water-mixture) twice a week. The *Artemia*-water-mixture was also added to the treatment tank during the 24 hour exposure to stimulate food-intake.

### Microplastic treatment

For exposure, corals were relocated from the four hosting tanks to the exposure tanks for 24 hours every two weeks (Fig. 1). The two treatment tanks without microplastic served as a procedural control for stress introduced by the movement from hosting tank to treatment tank.

Microplastic particles used in the experiment consist of pristine PET purchased from Goodfellow Cambridge Limited. PET was chosen because of its wide abundance in ocean waters, its high density (1.38 g cm^-3^) that allows it to settle on the coral surfaces, and as it is not toxic, thus reducing the stress parameters that affect the coral. The particles have an irregular shape and their size distribution ranges from ~ 5 to ~ 500 μm. The concentration of microplastic particles in the exposure setup was maintained at 0.5 g L^-1^ seawater. The microplastic particles were kept moving in the water column using a circulating streaming pump setup. Circulation decreased over time as an effect of the formation of biofilms on the particles causing increased particle weight with time. This biofilm formation, as well as a minimal loss of plastic material due to ingestion and adhesion, necessitated an exchange of microplastic particles in the exposure setup every two to three exposures.

### Seawater chemistry and maintenance of hosting tanks

Water chemistry conditions in the tanks were regularly monitored and kept stable during the duration of the experiment with a 10% water exchange weekly. Nitrate, phosphate and alkalinity were measured using microtiter plate readers (TECAN, Infinite 200 PRO F, Switzerland). Magnesium and calcium concentrations were determined using Inductively Coupled Plasma—Optical Emission Spectrometry (ICP-OES) following standard protocols for seawater analysis; some measurements were completed by hand using a NYOS titration kit for the coral reef aquaria.

Mean tank water temperature was kept at 24.97 ± 0.77 °C with a practical salinity of 34.8 ± 0.38. In a natural setting, phosphate and nitrate are introduced in the corals’ living environment by organic matter produced by reef fish. This aquaria setup lacked the introduction of organic matter as a result of the experimental design, thus requiring supplements provided artificially to enhance the stability of the system. Therefore, for the last six weeks of the experiment, the nutrition components Sangokai Nutri complex providing additional phosphate and Sangokai Nutri complete providing both nitrate and phosphate were added directly into the water column of the resting tanks. Both additives were supplied in a concentration of 1 ml per 100 L twice a week. Nitrate and phosphate levels were kept below detection level (NO_3_^-^:0.08 μM; PO_4_^3-^: 0.07 μM). The water chemistry parameters directly influencing calcification were kept stable throughout the experiment (Mg^2+^: 1300 mg L^-1^, Ca^2+^: 410 mg L^-1^, Alkalinity: 8 °dH = 1.429 mmol/l). During exposure, the seawater temperature was maintained at 25 °C using a Shego thermostat and a titanium tube heating element. Salinity and pH levels of the exposure setup were monitored before and after the exposure to ensure consistent conditions. Additional information regarding the seawater chemistry and physical parameters are given in the Supplementary Materials (Table S1-3).

Lighting for the hosting and the exposure tanks was implemented by Aqua Illumination Hydra fifty-two HD LED lamps with a sunlight and moonlight simulation program (11:13 h day/night ratio) with synchronised light spectra and intensities across all tanks. Light levels were aligned to the tanks of coral parent colonies peaking every day with an intensity of around 200 μmol s^-1^ m^-2^.

Each tank held several snails of the genus *Trochus* and also one shrimp (*Lysmata amboiensis*) to ensure a clean and healthy state of the aquaria. The *Lysmata amboiensis* were fed twice a day with freeze-dried food.

### Sample preparation

At the end of the five month experiment, the coral fragments were taken from the tanks and processed for further analysis. The corals from the control runs were collected first to avoid cross-contamination with the samples exposed to the microplastics. Within those groups, the specimens of the four species were processed in separate batches to avoid contamination across those species. Organic material was removed from the corals with a high-pressure painting sprayer pistol filled with 18.2 MΩ milli-Q water. The vertical growth of the corals (linear extension) was determined by comparing images taken before and after the experimental phase. The results were compared to species-specific growth rates based on vertical linear extension of *S. hystrix*, *P. damicornis* and *A. valida* calculated from the Coral Trait Database (Version 1.1.1) (Madin et al. 2016), while no such information was available for *M. capricornis*. Thin sections of the coral skeletal samples were prepared from the outermost 3–5 cm of the branching tips. The thin sections were stained for 30 s in a 0.2% HCl and 0.2 g/100 ml Alizarin red S solution (Warne 1962) to differentiate coral skeletal aragonite (stained red) from microplastic particles (not stained). The thin sections were sputter coated with gold for scanning electron microscopy (SEM) and energy dispersive X-ray spectroscopy (EDX) analyses. In order to validate our observational method, calibration thin sections of pristine plastic particles (PET) and pieces of coral skeleton were prepared (Fig. 4a).

### Microscopical examination of the skeletons

For light microscopy, a Keyence VHX 5000 was used to capture high-resolution images of the thin sections. For confirming the identification of skeletal materials, microplastic particles and embedding epoxy resin, EDX analytical technique was performed using the Tescan Vega XMU Scanning Electron Microscope. Brightness and contrast were post-processed in some images with Affinity Photo (Version 1.7.2) to reach optimal comparability across multiple thin sections. ImageJ image processing program was used for size measurements of microplastic particles (Version 1.52q) (Schneider et al. 2012).

## Results

### Visual observations during experimentation

**Fig. 2:**
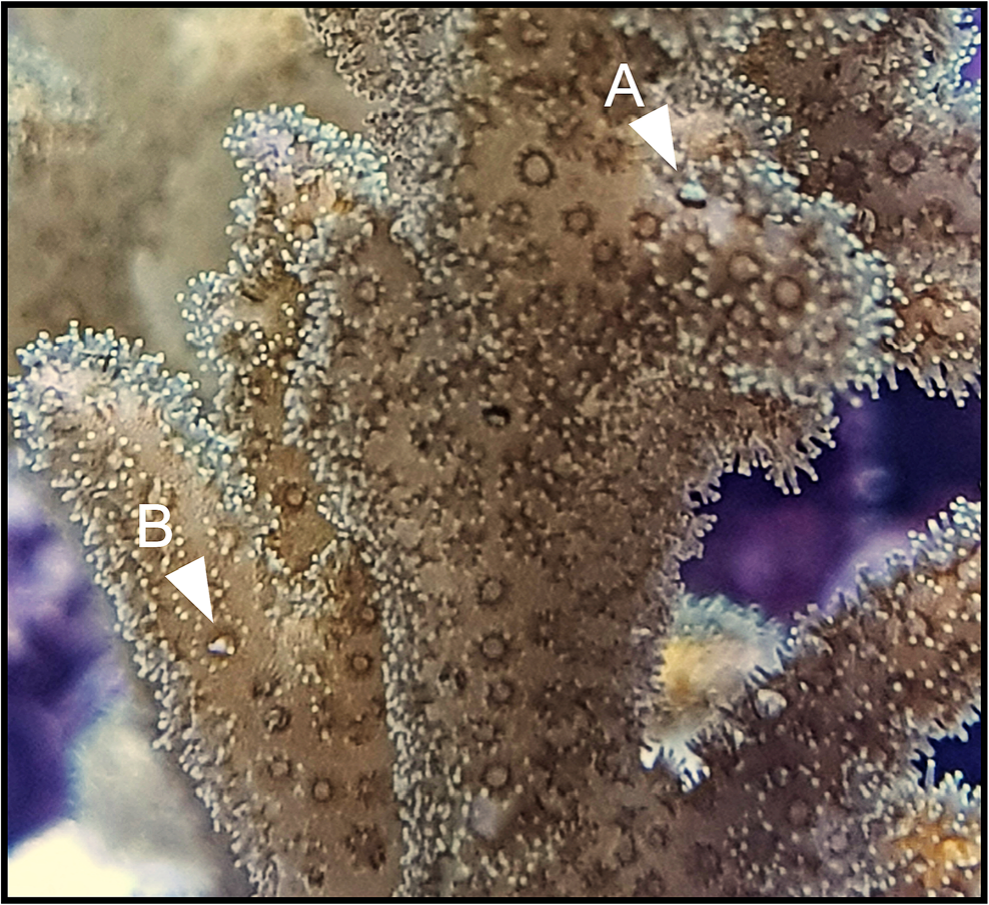
Ingestion and egestion of microplastic particles observed during and after microplastic exposure. White arrows indicate polyps that ingested microplastics. Polyp is closed after ingestion of microplastics (A). Polyp is opened; microplastic particle is observed inside the polyp mouth (B)

**Fig. 3:**
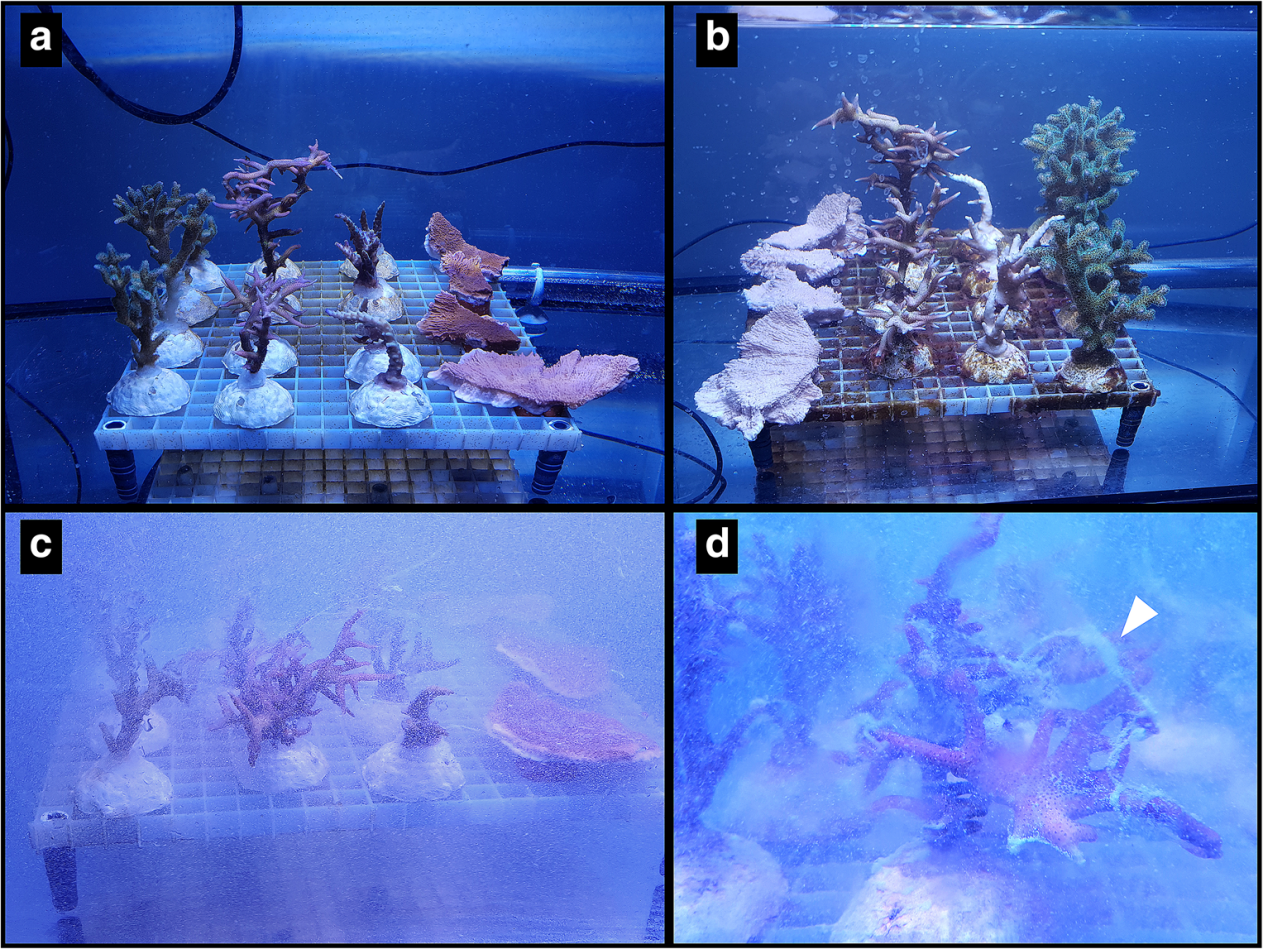
Visual observations during experimentation. **a** Coral control tank during the first round of exposure. **b** Coral control tank during the last round of exposure. A direct comparison of **a** and **b** visualizes extent of coral growth over the five month duration of the experiment. **c** Exposure tank during microplastic exposure. Notable increase in mucus production is visible when compared to **a** and **b**. **d** Extreme mucus production (white arrow) of *S. hystrix* inside the experimental setup during the first hours of exposure to microplastic

All corals survived the experiment in both, exposure or control treatments. Colour intensities of all corals faded equally for control and exposure, but no bleaching was observed. During the microplastic exposures, the corals were observed to ingest and egest particles (Fig. 2). Growth of a few centimetres was observed across species in images taken before and after experiment (Fig. 3a and b). Corals displayed increased mucus production compared to control corals during the first hours of exposure to microplastic particles (Fig. 3c and d). Inter-species differences in the amount of mucus expelled were observed with the highest amounts produced by *S. hystrix* (Fig. 3d). The lowest amounts of mucus were expelled by *M. capricornis*, while mucus production of *P. damicornis* and *A. valida* was slightly higher and comparable between the two species.

### Thin section analyses

**Fig. 4:**
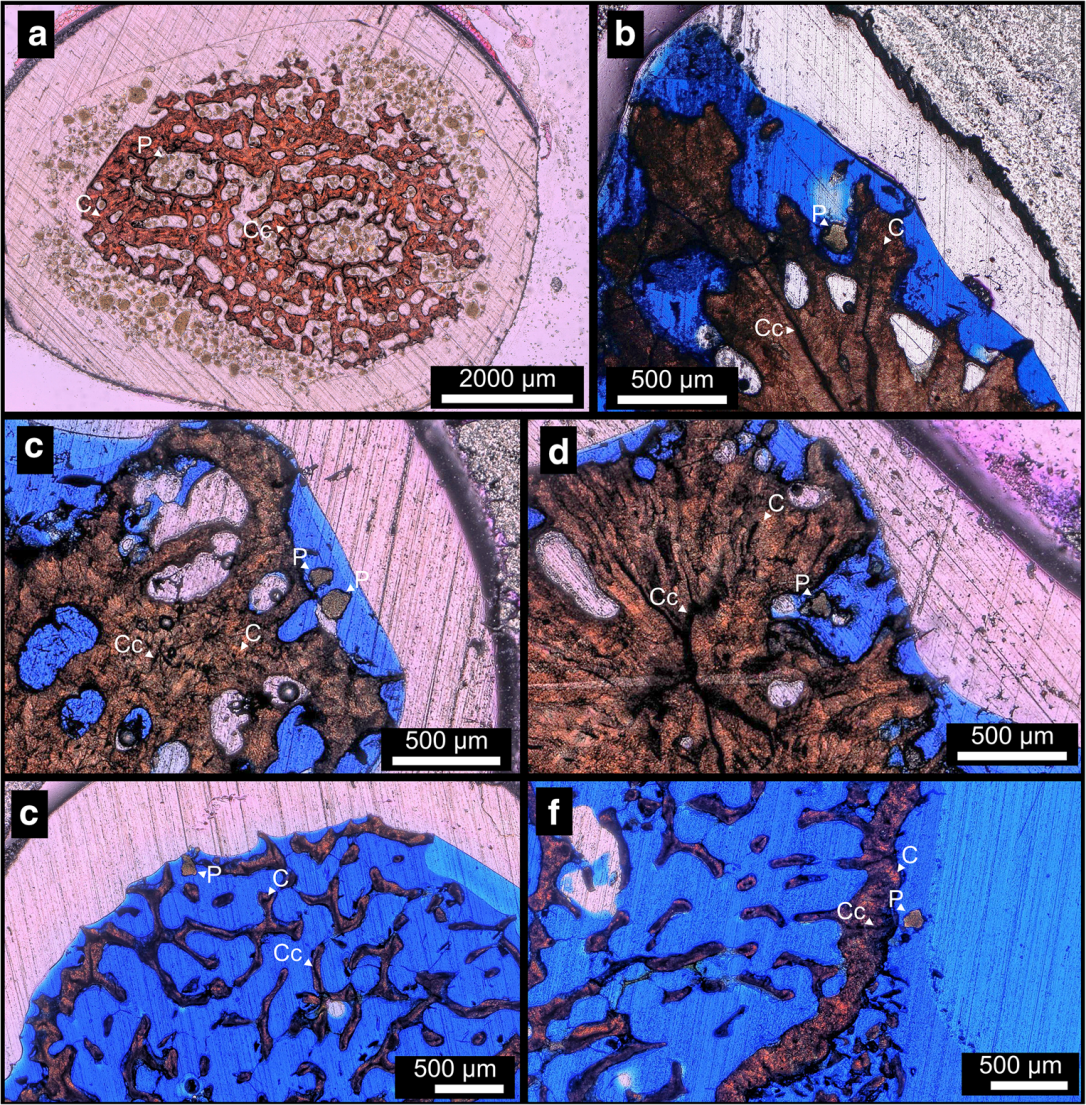
Microplastic particle and coral skeleton thin section comparison. Thin sections of **a** pristine plastic and coral skeleton particles as standards, and **b** of *S. hystrix* and *M. capricornis* fragments after exposure to microplastic particles. Aragonitic corals (C) were stained (red), while the stain did not affect plastic particles (P) that appear yellow-brown. Cc indicates the centre of calcification of corals. **c** Two unstained plastic particles located on the outer edge of *S. hystrix* skeleton, fine connections to the skeleton can be identified with polarized light microscopy. **d** Microplastic particle in the corallite cavity of *S. hystrix*. **e** Microplastic particle located close to the outer layer of *M. capricornis* skeleton. **f** Microplastic particle without direct visible connection to the skeleton found in a *M. capricornis* thin section

Thin sections of 32 coral fragments were inspected of which 13 are from the control runs and 19 from microplastic exposure treatments. In seven specimens of the exposure group, 10 particles interpreted as microplastic have been found to be associated (no visible encrustation) and incorporated (visible encrustation) in the skeletons of *S. hystrix* (*n* = 4) and *M. capricornis* (*n* = 3) (Fig. 4b–d). In contrast, no particles were identified in the specimens of *A. valida* and *P. damicornis* in the exposure treatment. Also, the control run specimens did not show any particles.

While there were clear visible structures in the coral skeleton (e.g. elongated crystals and centres of calcification), the microplastic particles exhibited a more random and homogenic crystallographic structure when inspected under a polarized light microscope (Fig. 4b–f). The microplastic particles identified in the thin sections of *S. hystrix* (Fig. 4b–d) and *M. capricornis* (Fig. 4e and f) are present mostly in the outermost polyp layer in close proximity to where the polyp-corallite interface would be. In *S. hystrix* (Fig. 4b–d), the particles were located on and in the outermost tips of the branches*.* In two *S. hystrix* sections (Fig. 4b), the integration of particles into the coral skeleton was obvious by a thin layer of carbonate having overgrown the particles (Fig. 4b), the other two *S. hystrix* thin sections did not display incorporation. Particles in *M. capricornis* (Fig. 4e and f) were present on top and inside the outer layer of the coral skeleton, sitting between the skeletal walls and septa, but with no visible encrustation happening. Those particles found in microplastic exposure thin sections of *S. hystrix* and *M. capricornis* had a mean length of 117 μm and a mean width of 82 μm.

### SEM/EDX analysis

**Fig. 5:**
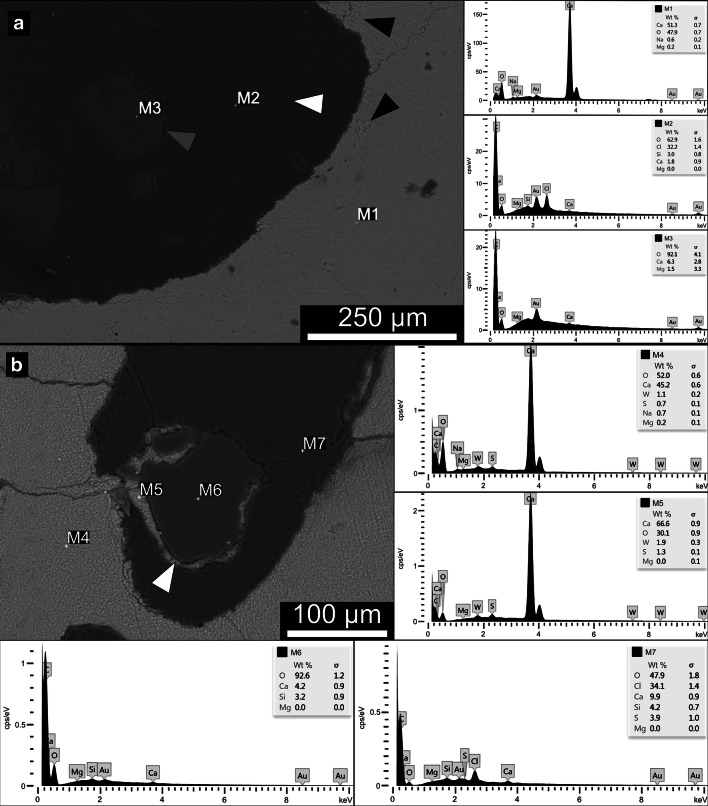
SEM images including point spectra of EDX measurements. **a** Artificially produced thin section created before the start of the experimentation. White arrow indicates surface structure of the epoxy resin induced by the cutting process. Black arrow indicates coral internal structure, individual aragonite crystals are visible. Three different measurements were taken (M1, M2, M3). All three measurements exhibit unique elemental spectra. M1 displays an increased amount of calcium (Ca) and oxygen (O). M2 displays an increased amount of oxygen (O) and a peak in chlorine (Cl). M3 displays an increased amount in oxygen (O) and does not have a peak in chlorine (Cl). M1 was a measurement of coral skeleton, M2 was a measurement of the epoxy resin and M3 was the measurement of microplastic particles. **b** Coral skeleton of *S. hystrix* revealing the possible mechanism of how the microplastic particles were held in place. White arrow indicates the carbonate layer forming around the microplastic particle. Four different measurements (M4, M5, M6, M7) were completed across the sample. M4 compared to M5 resolves a very similar pattern, with only a slight difference in oxygen (O) content was detected. M4 and M5 display high peaks in calcium (Ca). M6 displays a high peak in oxygen (O). M7 displays a high peak in oxygen (O) and a smaller peak in chlorine (Cl)

Backscattered electron microscopy (BSE) showed a clear difference between coral skeleton and the two types of polymers (PET and epoxy resin) while between the latter, only a subtle difference is present. In descending order, coral skeleton, microplastic particles, and epoxy resin differed from light grey to dark grey. Resulting from the curing process of the epoxy resin, a characteristic gap between resin and the other two materials was evident (Fig. 5a, grey arrow). The surface structure of the coral skeleton displayed visible areas with aragonite crystals (Fig. 5a, black arrow). A differentiation between epoxy and microplastic by their surface structure was not possible. Furrows in both polymer structures were results of the thin section preparation process originating from the low hardness of the polymer materials (Fig. 5a, white arrow). Furrows can also be seen in carbonate material, but with much less reprint.

EDX elemental analyses were performed on all seven thin sections from the PET-exposure treatment that contained plastic particles. Measurements were dominated by three different elemental spectra types similar to M1, M2 and M3 in the calibration thin section (Fig. 5a). The elemental spectra in this example (Fig. 5b) were labelled as M4 to M7. M4 and M5 are characterised by a high peak in calcium (Ca) and a smaller peak in magnesium (Mg) and oxygen (O) (Fig. 5b, panel M4 and M5). M6 is characterised by a high peak in oxygen (O) (Fig. 5b, panel M6). M7 is characterised by a small peak in oxygen (O) and the presence of chlorine (Cl) (Fig. 5b, panel M7). All particles previously identified by light microscopy are confirmed as microplastics based on the calibration with the artificially produced thin section. The BSE analysis of an encrusted particle (Fig. 5b, white arrow) shows that the microplastic particle is surrounded almost entirely by a 2–15 μm thick layer of carbonate material and set in the lower part of the corallite. In the other thin sections, particles were either situated in the corallite cavity of the outer branches, or on random locations attached to the outer skeletal portion.

### EDX analyses

The EDX detector was used to identify the characteristic elemental composition of the materials in the calibration thin section of microplastic particles next to coral skeletal aragonite embedded in epoxy resin (Fig. 5a). These three components could be clearly distinguished in EDX, the coral skeletal materials being characterised by calcium (Ca), magnesium (Mg), and oxygen (O) peaks (Fig. 5a, panel M1) while the characteristic profile of epoxy resin contains mostly chlorine (Cl), and oxygen (O) (Fig. 5a, panel M2), and for the microplastic particles, a high peak in oxygen (O) was observed (Fig. 5a, panel M3). The elemental spectra of the epoxy resin and the microplastics show minor traces of calcium (Ca) and magnesium (Mg).

The curve shape of the elemental analysis in the thin section of plastic exposed coral samples, analyses M4 and M5 (Fig. 5b, panel M4 and M5), shows high calcium (Ca) values similar to M1 (Fig. 5a, panel M1) i.e. the coral skeleton in the calibration thin section. M7 (Fig. 5b, panel M7) in the thin section of a microplastic exposed coral is identified as epoxy resin, by its high values in chlorine (Cl), similar to M2 (Fig. 5a, panel M2). Finally, M6 (Fig. 5b, panel M6) in the exposed thin section can be identified as microplastic particle by its similarity to M3 in the calibration thin section (Fig. 5a, panel M3) with its high values in oxygen (O) and the lack of chlorine (Cl). Additionally, elemental weight analysis (Table 1) yielded comparable values.

**Table 1:** Measurements of elemental point spectra in weight percentage (wt%)

Measurement in wt%		Ca	O	Cl	Mg	Na	Si	S	W	Sample
M1	Coral skeleton	51.27	47.88	-	0.21	0.64	-	-	-	Calibration thin section of plastics and coral skeleton (Fig. 5a)
M2	Epoxy resin	1.79	62.93	32.24	0	-	3.03	-	-	
M3	Plastic particle	6.33	92.12	-	1.55	-	-	-	-	
M4	Coral skeleton	45.24	51.98	-	0.23	0.74	-	0.75	1.07	Thin section of corals exposed to plastic (Fig. 5b)
M5	Coral skeleton	66.62	30.15	-	0.04	-	-	1.31	1.89	
M6	Plastic particle	4.21	92.64	-	0	-	3.15	-	-	
M7	Epoxy resin	9.91	47.85	34.1	0	-	4.23	3.91	-	

### Fibres encrusted by coral skeleton

**Fig. 6:**
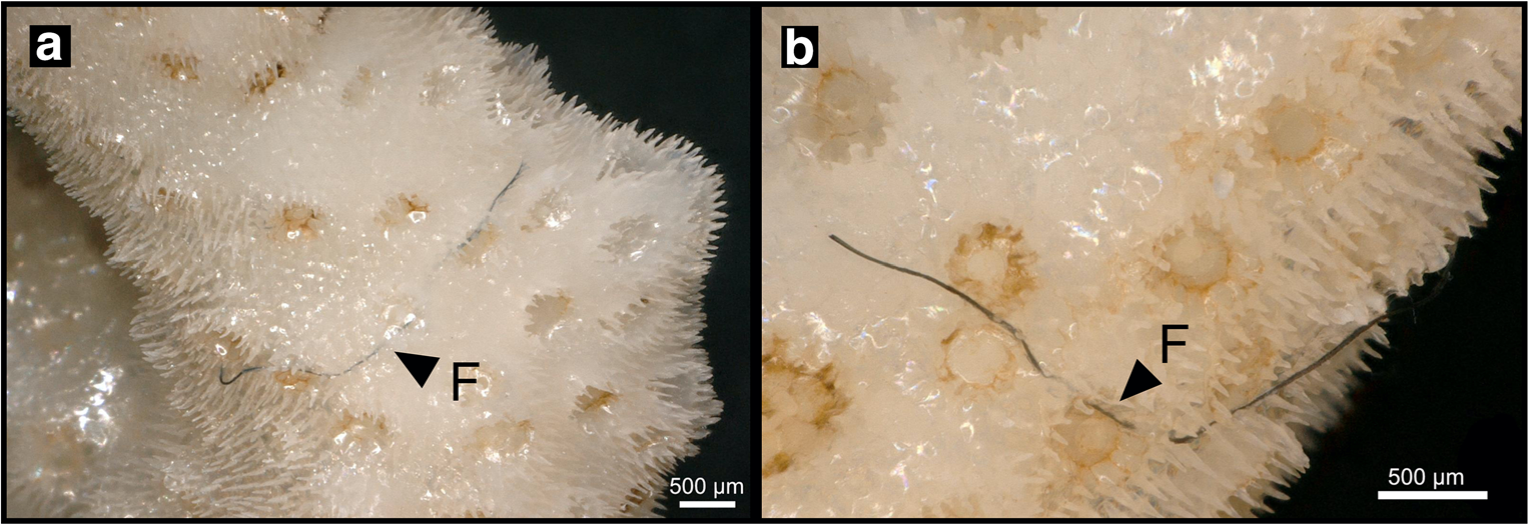
Microplastic fibre incorporation. **a** *S. hystrix* skeleton displaying an embedded microfibre (length: ~3861.44 μm) (F). **b** Partial entanglement of microfibre (length: ~3462.13 μm) (F). Fibres positioned across multiple corallites

Although fibres were not added to the experiment on purpose, fibres were found to be partially embedded in the coenosteum of two coral skeletons of *S. hystrix* (Fig. 6a, b). The fibres are ca. 3.5 to 4 mm long, have a black colour and were encrusted in close proximity to the corallites (Fig. 6a, b). SEM analyses revealed carbonate coating of the edges of microfibres.

## Discussion

The PET-exposure experiment shows a species-specific impact of microplastic particles on corals, namely, with *S. hystrix* and *M. capricornis* showing more frequent incorporation of particles. Additionally, *S. hystrix* displayed higher mucus production in the first hours of exposure compared to *P. damicornis, A. valida* and *M. capricornis* (Fig. 3d). This is in agreement with previous observations of species-specific variabilities in coral health responses to microplastics (Reichert et al. 2019). We speculate that the increase in mucus production is critical in the higher trapping rates of microplastics close to the polyps, thus elevating the probability of microplastic incorporation. The increased trapping as observed for *M. capricornis* may be connected to the flat morphology and larger surface area exposed to the settling particles. Settling on the coral surface observed during treatment is thought to increase the availability of particles for the ingestion by individual polyps. In addition, the decrease in flow rate due to the mounting of *M. capricornis* on the grid likely trapped further microplastic particles underneath the coral, as they were not only found on top but also at the bottom side of the skeleton.

The size of most of the microplastic particles observed to be incorporated by corals corresponds to the preferred size of particulate organic matter taken up by scleractinian corals (~100 μm) (Mills, 2004). Other studies have shown that corals favour the ingestion of pristine microplastic particles over the original food source of the same size and biofouled plastic particles (Allen et al. 2017; Reichert et al. 2018, 2019; Rotjan et al. 2019; Axworthy and Padilla-Gamiño 2019). This behaviour is thought by Allen et al. (2017) to have been triggered by the chemical composition of the microplastic particles. Uptake subsequently led to gut blockage and false satiation, a depletion of energy levels and ultimately to death in the form of bleaching (Wright et al. 2013; Watts et al. 2015; Hall et al. 2015; Biquand et al. 2017; Allen et al. 2017; Okubo et al. 2018; Hankins et al. 2018; Rotjan et al. 2019; Syakti et al. 2019). A recent study revealed that ingested plastic microspheres were incorporated into the cells of the mesenterial filaments of specimens of the anthozoan genus *Exaiptasia* after entering the gastrovascular cavity, while migration into the tissue interface is thought to depend on particle size (Okubo et al. 2020).

The observations presented here indicate that corals with larger polyp diameters such as *A. valida* and *P. damicornis* can potentially better control uptake and egestion and thus protect themselves against microplastic pollution than *S. hystrix* and *M. capricornis* with their smaller polyps. This is in agreement with previous observations that have shown that large polyp species are less affected by microplastic pollution than smaller polyp species (Hankins et al. 2018). Smaller polyps lose their potential to ingest nutritional prey while they are busy coping with ingested microplastic particles (Rotjan et al. 2019). Moreover, particles with a bigger diameter may become lodged inside a coral’s gastrovascular space more easily.

After ingestion, if microplastic particles pass into the coral gut cavity, they are likely overgrown by tissue and then later encrusted in the tissue by carbonate skeleton material. The encrustation of microplastic particles in the skeleton of *S. hystrix* supports the overgrowth hypothesis suggested by Reichert et al. (2018), where plastic particles were overgrown by coral tissue as a result of necrosis. In our experiment, the two encrusted particles are located at the bottom of a corallite in the area of the lower gastrointestinal tract of the polyp (Figs. 4b and 5b). The particles are surrounded by aragonite, in one case connected to the skeleton. The mechanism of encrustation is interpreted as the formation of a new basal plate around the particle. A similar incorporation pattern was shown for sediments of non-carbonate origin trapped in between dissepiments and basal plates of *Solenastrea hyades*, *Pavona gigantea* and *Montastrea annularis* (Barnard et al. 1974)*.*

Another mode of overgrowth and encrustation is seen around fibres that were found incorporated in the coral skeleton (Fig. 6a and b). These fibres are likely sourced by prevailing airborne contamination during the experiment in the open tank system, or might be introduced in the system by the sea salt used. Sea salt produced for human consumption was recently shown to contain microplastics (Peixoto et al. 2019); thus, it is likely that sea salt for aquarium use produced under less stringent controls than for human consumption might contain microplastic particles and fibres. These fibres were found encrusted in the coenchym areas in between corallites. A plausible mechanism for fibre encrustation is that after settling on the outer edges of the polyps, they got entangled between polyps beyond their reach of active removal. Similar entanglement and overgrowth of monofilament fishing lines have previously been observed (Smith and Hattori 2008).

**Fig. 7:**
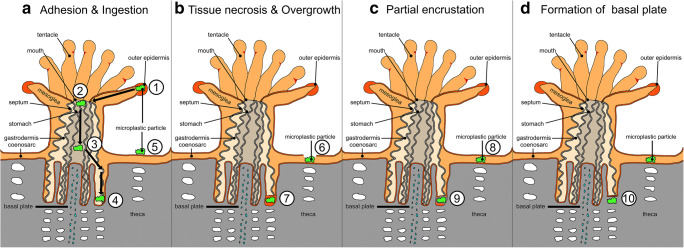
Possible microplastic incorporation scenario. **a** (1) Microplastic particle becomes adhesive to the outer epidermis area of the coral tentacle. (2) Food response is triggered and the particle is transported to the mouth of the polyp. (3) Ingestion begins and the particle is transported into the gastrovascular cavity. (4) The microplastic particle becomes stuck inside the gastral area. (5) Adhesion to the coenosarc as a possible mechanism of attachment. **b** (6) Tissue necrosis occurs and particles migrate into the tissue. (7) Tissue healing process starts and the particle is overgrown by tissue material. **c** (8) Tissue healing process is completed. The particle is fully surrounded by coral tissue. (9) Partial overgrowth by aragonite is induced. Aragonite crystals form a layer surrounding the foreign body, later connected to the theca. **d** (10) The formation of a new basal plate surrounding the microplastic particle by encrustation is complete

The overgrowth by coral tissue and skeleton may be a result of a temporal tissue necrosis resulting from microplastic adhesion (Reichert et al. 2018; Martin et al. 2019; Corona et al. 2020). Following necrosis, the damaged tissue is recolonised by intact tissue, partially encrusting the foreign fibre or particle (Fig. 6a and b) and incorporating it into the skeletal material (Fig. 4b). The microplastic particles or fibres become adhesive to the coral surface structure, either by attachment to the tentacle or the coenosarc (Fig. 7a). When caught by a tentacle, the immediate food response of the coral is triggered and ingestion begins (Fig. 7a), as also described earlier (Allen et al. 2017). Upon reaching the gastral area, plastic particles become stuck inside the gastrovascular cavity inducing tissue necrosis. Tissue necrosis usually occurs directly in connection with surface exposure to microplastic (Reichert et al. 2018). This process is followed by clonal tissue overgrowth as healing mechanism of the tissue damage (Fig. 7b). Following the complete tissue overgrowth, partial encrustation by aragonite material takes place (Fig. 7c) as aragonite crystals form around the foreign body and induce the formation of a new basal plate (Fig. 7c). When the formation of a new basal plate is completed, the microplastic material is fully embedded in the coral skeleton (Fig. 7d).

Although PET concentrations of 0.5 g L^-1^ in the experiment were above concentrations occurring in the environment (Reisser et al. 2013; Syakti et al. 2017; Ding et al. 2019; Jensen et al. 2019), the observed effects may increase and accumulate over the lifetime of a coral colony. Concentrations in polluted environments on a reef flat have been reported to reach some 12.2 items L^-1^ (Ding et al. 2019). It has been shown that rejection, ingestion, and egestion are energy consuming (Reichert et al. 2019) as are the increased mucus production and active particle removal (Wild et al. 2004). In the end, however, the low portion of microplastic particles incorporated compared to the high concentration of exposure indicates that corals are very efficient in removing and rejecting microplastics, *P. damicornis* and *A. valida* even more successful than *S. hystrix* and *M. capricornis*.

In a future that is expected to see increased plastic littering, coral reef environments may become a major sink for microplastics by adhesion, ingestion, and skeletal incorporation (Reichert et al. 2018; Martin et al. 2019; Corona et al. 2020). It has been shown that short-term exposure to microplastics results in minor to almost no effect on the coral calcification rates (Hankins et al. 2018). However, no information is as yet available on the long-term effects of exposure to microplastics. We speculate that because species with larger polyp diameters may be less impacted than species with smaller polyp diameters, a shift in species composition might result from long-term exposure to high loads of microplastic pollution. Our results additionally imply that microplastics incorporated into the coral skeletal materials might enter the food chain of grazers such as corallivorous fish, and ultimately appear in the human diet (Andrady 2011). The particles may be released again in the water column after bioerosive processes such as Parrotfish bites (Hutchings 1986).

## Supplementary Information

ESM 1 (PDF 149 kb)

## Data Availability

The datasets used and analysed during the current study are available from the corresponding author on reasonable request.
