# Supplementary material for: Scleractinian corals incorporate microplastic particles: identification from a laboratory study
Source: Environ Sci Pollut Res Int. 2021 Mar 15;28(28):37882–93. doi: 10.1007/s11356-021-13240-x (PMC8302493; doi:10.1007/s11356-021-13240-x)
Supplement: Supplementary file 1 — (PDF 149 kb) [file 11356_2021_13240_MOESM1_ESM.pdf]

Supplementary material for

Scleractinian corals incorporate microplastic particles: Identification from a laboratory study

Florian Hierl [1,2,*], Henry C. Wu [1], Hildegard Westphal [1,2]

[1] Leibniz Centre for Tropical Marine Research (ZMT), Fahrenheitstraße 6, Bremen D-28359, Germany

[2] Faculty of Geosciences, University of Bremen, Bremen, Germany

*Correspondence to: florian.hierl@leibniz-zmt.de (F. Hierl)

This PDF file includes:

Supplementary Tables 1 to 3

Supplementary Table S1. Resting tank water chemistry.

Weekly measurements of Phosphate (PO_4^{3-}, µmol/L), Nitrate (NO_3^-, µmol/L), Carbonate Hardness (KH, ºdH), Calcium (Ca, ppm), Magnesium (Mg, ppm), Potassium (K, ppm) and Strontium (Sr, ppm). ET09 = Exposure 1 (E1), ET10 = Exposure 2 (E2), ET11 = Control 3 (C3), ET12 = Control 4 (C4); bDL= Below Detection Limit; N/A = Data Not Available

Tank name	Date [yyyymmdd]	c(NO_3^-) calc. [µmol/L] (+c(NO2))	c(PO_4^{3-}) calc. [µmol/L]	KH [°dH]	Ca [ppm]	Mg [ppm]	K [ppm]	Sr [ppm]
ET_09	20180830	bDL	0.2	9.2	380	1260	N/A	N/A
ET_10	20180830	bDL	0.2	9.2	390	1230	N/A	N/A
ET11	20180830	0.5	0.6	9.7	400	1260	N/A	N/A
ET12	20180830	0.1	0.1	9.3	400	1260	N/A	N/A
ET_09	20180905	0.3	bDL	8.9	474	1296	N/A	N/A
ET_10	20180905	0.5	bDL	8.3	455	1313	N/A	N/A
ET11	20180905	0.5	bDL	8.6	448	1288	N/A	N/A
ET12	20180905	2.9	bDL	8.5	446	1293	N/A	N/A
ET_09	20180913	bDL	0.2	8.3	N/A	N/A	N/A	N/A
ET_10	20180913	0.2	0.1	10.0	N/A	N/A	N/A	N/A
ET11	20180913	bDL	bDL	8.8	N/A	N/A	N/A	N/A
ET12	20180913	bDL	0.9	8.4	N/A	N/A	N/A	N/A
ET_09	20180926	bDL	bDL	6.4	427	1179	N/A	N/A
ET_10	20180926	bDL	bDL	6.5	419	1179	N/A	N/A
ET11	20180926	bDL	bDL	7.0	418	1165	N/A	N/A
ET12	20180926	bDL	bDL	6.5	419	1180	N/A	N/A
ET09	20181002	0.45	0.12	7.4	440	1409	383	4.40
ET10	20181002	0.39	0.12	7.1	444	1414	385	4.30
ET11	20181002	0.85	0.04	7.0	442	1418	389	4.40
ET12	20181002	1.48	bDL	7.4	430	1399	386	4.40
ET_09	20181009	2.88	bDL	6.6	432	1352	369	4.3
ET_10	20181009	bDL	bDL	6.4	435	1366	370	4.3
ET11	20181009	bDL	bDL	6.5	435	1379	372	4.4
ET12	20181009	bDL	0.11	6.7	431	1371	375	4.3
ET09	20181017	0.76	N/A	7.4	421	1337	372	4.3
ET10	20181017	3.77	N/A	7.4	420	1350	377	4.2
ET11	20181017	bDL	N/A	7.6	418	1329	369	4.3
ET12	20181017	5.49	N/A	7.2	416	1313	376	4.3
ET09	20181023	0.44	0.11	7.3	405	1303	356	4.10
ET10	20181023	0.91	0.16	7.2	394	1300	340	4.00
ET11	20181023	0.87	bDL	7.2	402	1305	351	4.10
ET12	20181023	1.92	bDL	7.4	396	1273	341	4.20
ET09	20181030	bDL	0.18	7.4	400	1285	338	4.10
ET10	20181030	bDL	bDL	6.7	410	1294	343	4.00

ET11	20181030	bDL	bDL	7.1	406	1298	344	4.10
ET12	20181030	bDL	bDL	7.4	406	1288	344	4.10
ET09	20181106	bDL	bDL	7.6	415	1237	354	3.30
ET10	20181106	bDL	bDL	7.1	426	1206	356	5.50
ET11	20181106	bDL	bDL	7.2	424	1191	355	5.40
ET12	20181106	bDL	bDL	7.3	409	1211	340	5.10
ET09	20181113	bDL	bDL	7.5	379	1274	314	3.9
ET10	20181113	bDL	bDL	7.1	377	1301	329	3.8
ET11	20181113	bDL	bDL	7.5	378	1299	333	3.9
ET12	20181113	bDL	bDL	7.7	379	1302	335	3.9
ET09	20181121	bDL	bDL	6.8	401	1316	353	4
ET10	20181121	bDL	bDL	6.4	398	1321	359	3.9
ET11	20181121	bDL	bDL	7.1	400	1324	363	4
ET12	20181121	bDL	bDL	6.8	399	1317	358	4
ET09	20181128	0.3	0.00	7.1	396	1249	284.7	3.8
ET10	20181128	0.00	0.00	7.5	390	1261	276.2	3.7
ET11	20181128	0.00	0.00	7.4	404	1277	312.9	3.9
ET12	20181128	0.00	0.13	7.7	412	1289	309.3	3.9
ET09	20181204	bDL	bDL	6,9	400	1317	339	3,9
ET10	20181204	bDL	bDL	6,2	402	1289	346	3,8
ET11	20181204	bDL	bDL	6,8	399	1288	345	3,9
ET12	20181204	bDL	bDL	6,6	410	1290	352	4,0
ET09	20181212	bDL	bDL	7,6	411	1303	359	4,0
ET10	20181212	bDL	bDL	6,8	408	1310	356	3,9
ET11	20181212	bDL	bDL	7,4	410	1285	354	4,0
ET12	20181212	bDL	0,08	7,0	409	1298	358	4,0
ET09	20181218	bDL	bDL	7,1	397	1287	346	3,9
ET10	20181218	bDL	bDL	7,1	396	1286	348	3,8
ET11	20181218	bDL	bDL	7,3	398	1282	348	3,9
ET12	20181218	bDL	bDL	7,7	395	1266	351	3,9
ET09	20181227	N/A	N/A	8	370	1290	N/A	N/A
ET10	20181227	N/A	N/A	8	390	1320	N/A	N/A
ET11	20181227	N/A	N/A	8	430	1320	N/A	N/A
ET12	20181227	N/A	N/A	8	390	1320	N/A	N/A
ET09	20190102	N/A	N/A	7	390	1320	N/A	N/A
ET10	20190102	N/A	N/A	8	400	1290	N/A	N/A
ET11	20190102	N/A	N/A	7	380	1320	N/A	N/A
ET12	20190102	N/A	N/A	7	430	1290	N/A	N/A
ET09	20190107	bDL	bDL	7,7	428	1308	388	4,0
ET10	20190107	bDL	bDL	6,5	419	1294	393	3,9
ET11	20190107	bDL	bDL	7,5	425	1286	395	4,1
ET12	20190107	bDL	bDL	7,7	414	1283	387	4,0
ET09	20190114	bDL	bDL	7,8	430	1303	385	4,1

ET10	20190114	bDL	bDL	7,4	418	1296	381	4,0
ET11	20190114	bDL	bDL	7,3	421	1293	380	4,2
ET12	20190114	bDL	bDL	7,4	415	1281	384	4,1
ET09	20190121	bDL	bDL	6.7	408	1264	361.3	3.9
ET10	20190121	bDL	bDL	6.5	417	1264	371.7	3.9
ET11	20190121	bDL	bDL	6.9	416	1265	372.2	4.1
ET12	20190121	bDL	bDL	6.8	415	1281	366.6	4
ET09	20190128	5.687	0	6.1	411	1241	364.7	N/A
ET10	20190128	0.506	0	5.8	406	1175	357	N/A
ET11	20190128	0.363	0	6	408	1167	359.3	N/A
ET12	20190128	2.293	0	5.8	415	1182	369.8	N/A
ET09	20190204	18.938	0.1	5.2	419	1296	369	4
ET10	20190204	6.53	1.7	5.1	424	1354	370	3.9
ET11	20190204	7.828	8.6	4.8	420	1335	364	4.1
ET12	20190204	11.99	5.9	5.1	411	1326	360	4
ET09	20190211	12.878	0.07	8.8	402	1269	355.2	N/A
ET10	20190211	0.367	0.08	8.9	402	1295	359.1	N/A
ET11	20190211	2.802	0.09	8.7	401	1303	328.8	N/A
ET12	20190211	4.4	0.14	8.9	393	1280	349.1	N/A
ET09	20190219	8.081	bDL	8	399	1267	346	3.9
ET10	20190219	bDL	bDL	7.2	404	1276	355	3.9
ET11	20190219	bDL	bDL	8.1	407	1275	353	4
ET12	20190219	bDL	bDL	7.9	399	1293	352	3.8
ET09	20190225	6.099	0.1	7.2	400	1277.4	347.1	3.9
ET10	20190225	bDL	0.08	7.2	395	1245.7	338.3	3.9
ET11	20190225	bDL	0.08	7.1	395	1280	338.9	3.9
ET12	20190225	bDL	0.11	6.8	398	1283.2	347.1	3.8
ET09	20190304	8.829	0.07	7.4	415	1305.5	357.2	4.1
ET10	20190304	bDL	0.07	7.4	420	1324.2	355.3	4.2
ET11	20190304	1.299	0.18	7.2	421	1319.4	358.6	4.1
ET12	20190304	0.875	0.07	7.3	418	1308.5	354.9	4
ET09	20190311	4.324	0.03	8	434	1353	333	4.3
ET10	20190311	bDL	0.04	7.8	436	1369	304	4.3
ET11	20190311	1.43	0.07	8	439	1359	354	4.3
ET12	20190311	bDL	0.09	7.9	429	1345	312	4.1

Supplementary Table S2. Resting tank parameters.

pH (NBS Scale), Temperature (ºC), Salinity (S_P), and Oxygen (mg/L) content measurements taken twice a week from the resting tanks in addition to the continuous monitoring of fixed installed probes. ET09 = Exposure 1 (E1), ET10 = Exposure 2 (E2), ET11 = Control 3 (C3), ET12 = Control 4 (C4)

ET 9

**Date**	**pH**	**T**	**Salinity**	**O2**		
[dd.mm.yyyy]		[°C]		[mg/l]	[%]	[mbar]
26.10.2018	8,251	24.3	35.0	8.35	102.7	208
29.10.2018	8,236	25.1	34.8	8.34	101.5	208
02.11.2018	8,215	25.0	35.0	8.30	100.6	207
05.11.2018	8,215	25.1	34.8	8.40	101.8	209
09.11.2018	8,203	25.0	34.8	8.38	102.1	209
12.11.2018	8,197	25.0	34.8	8.42	102.3	210
16.11.2018	8,168	25.1	34.8	8.55	102.0	213
19.11.2018	8,199	25.0	35.0	8.44	101.6	211
23.11.2018	8,247	26.8	35.0	8.19	101.7	210
26.11.2018	8,190	21.5	34.8	8.98	101.5	210
30.11.2018	8,202	24.0	34.5	8.52	102.2	210
03.12.2018	8,224	24.1	34.9	8.35	101.6	206
07.12.2018	8,318	24.0	34.2	8.39	101.2	207
10.12.2018	8,325	24.0	34.2	8.54	101.5	209
14.12.2018	8,306	23.9	34.2	8.64	102.1	213
17.12.2018	8,280	24.3	34.2	8.47	101.4	210
21.12.2018	8,321	23.9	34.1	8.37	101.7	205
27.12.2018	8,287	24.4	34.1	8.64	101.8	212
07.01.2019	8,296	24.2	34.5	8.61	101.5	213
11.01.2019	8,261	24.1	34.6	8.52	101.2	210
14.01.2019	8,326	24.7	34.5	8.45	102.5	210
18.01.2019	8,300	25.1	34.7	8.35	101.0	208
21.01.2019	8,272	24.8	34.5	8.51	102.4	213
25.01.2019	8,242	24.6	34.5	8.38	100.7	207
28.01.2019	8,237	25.3	34.4	8.13	101.9	203
01.02.2019	8,263	25.1	34.5	8.18	101.6	204
04.02.2019	8,253	25.1	34.4	8.45	101.5	211
08.02.2019	8,310	25.2	34.5	8.27	101.1	206
11.02.2019	8,322	25.2	34.4	8.23	100.4	205
15.02.2019	8,220	24.1	34.5	8.68	101.7	214
18.02.2019	8,224	24.7	34.4	8.42	100.6	208
21.02.2019	8,397	24.1	34.4	8.69	102.9	214
25.02.2019	8,251	23.2	34.4	8.84	101.4	214
01.03.19	8,340	24.3	34.4	8.53	102.6	211

04.03.19	8,361	24.4	34.4	8.17	101.2	202
06.03.19	8,363	24.3	34.4	8.44	103.0	209

ET 10

**Date**	**pH**	**T**	**Salinity**	**O2**		
[dd.mm.yyyy]		[°C]		[mg/l]	[%]	[mbar]
26.10.18	8,223	24.2	35.0	8.36	102.1	208
29.10.18	8,222	25.1	34.8	8.35	101.2	208
02.11.18	8,216	25.0	34.9	8.31	101.0	208
05.11.18	8,200	25.0	34.8	8.37	101.5	208
09.11.18	8,206	25.1	34.8	8.42	102.0	209
12.11.18	8,185	25.1	34.8	8.41	102.1	209
16.11.18	8,175	25.1	34.8	8.54	102.0	213
19.11.18	8,198	25.1	35.0	8.47	101.4	211
23.11.18	8,244	26.7	35.2	8.17	101.5	209
26.11.18	8,180	21.6	34.8	9.01	102.1	211
30.11.18	8,179	24.4	34.7	8.50	102.3	210
03.12.18	8,190	24.3	34.8	8.37	101.9	206
07.12.18	8,319	24.1	34.2	8.44	101.7	208
10.12.18	8,313	24.3	34.2	8.48	101.5	209
14.12.18	8,312	24.0	34.1	8.69	102.2	213
17.12.18	8,287	24.4	34.1	8.54	101.3	209
21.12.18	8,331	24.0	34.2	8.38	101.9	206
27.12.18	8,274	24.3	34.1	8.59	102.1	213
07.01.19	8,210	24.3	34.6	8.68	102.2	214
11.01.19	8,244	24.4	34.6	8.52	101.9	211
14.01.19	8,330	24.7	34.5	8.52	102.9	210
18.01.19	8,291	25.2	34.5	8.33	100.8	208
21.01.19	8,270	25.0	34.5	8.49	102.7	213
25.01.19	8,249	24.6	34.5	8.38	100.5	207
28.01.19	8,232	25.4	34.4	8.09	101.5	203
01.02.19	8,262	25.2	34.5	8.15	101.0	203
04.02.19	8,247	25.2	34.5	8.41	101.0	210
08.02.19	8,292	25.2	34.5	8.24	100.4	205
11.02.19	8,306	25.3	34.5	8.20	100.4	205
15.02.19	8,201	24.2	34.5	8.73	102.4	215
18.02.19	8,207	24.7	34.4	8.37	100.0	207
21.02.19	8,379	24.3	34.4	8.74	103.2	215
25.02.19	8,223	23.3	34.4	8.88	101.9	215
01.03.19	8,319	24.4	34.5	8.54	102.4	211
04.03.19	8,338	24.7	34.5	8.15	101.5	203
06.03.19	8,422	23.9	33.9	8.61	104.7	214

ET 11

Date	pH	T	Salinity	O2		
[dd.mm.yyyy]		[°C]		[mg/l]	[%]	[mbar]
26.10.18	8,229	24.4	35.0	8.33	102.3	208
29.10.18	8,226	24.9	34.9	8.36	101.2	208
02.11.18	8,213	25.2	35.0	8.33	100.7	207
05.11.18	8,205	25.0	34.9	8.40	101.8	209
09.11.18	8,213	24.9	35.0	8.37	101.8	209
12.11.18	8,205	24.8	35.0	8.41	101.9	209
16.11.18	8,165	24.8	34.9	8.61	101.9	213
19.11.18	8,197	24.7	35.2	8.55	101.8	211
23.11.18	8,246	26.9	35.4	8.17	102.3	211
26.11.18	8,186	22.0	34.9	8.92	101.9	211
30.11.18	8,181	24.6	34.8	8.47	101.8	208
03.12.18	8,200	24.4	35.0	8.39	102.0	206
07.12.18	8,305	24.3	34.3	8.36	101.4	206
10.12.18	8,312	24.3	34.2	8.52	101.6	209
14.12.18	8,289	24.0	34.2	8.63	102.0	212
17.12.18	8,268	24.0	34.2	8.54	101.1	209
21.12.18	8,307	24.2	34.2	8.30	101.7	205
27.12.18	8,276	24.6	34.3	8.62	101.8	212
07.01.19	8,216	24.5	34.7	8.61	101.9	213
11.01.19	8,233	24.6	34.6	8.53	101.6	211
14.01.19	8,311	24.2	34.5	8.60	102.6	210
18.01.18	8,297	25.1	34.6	8.43	101.7	209
21.01.19	8,300	24.8	34.6	8.58	102.7	213
25.01.19	8,224	23.8	34.5	8.51	100.7	207
28.01.19	8,209	24.9	34.6	8.20	101.7	203
01.02.19	8,226	24.7	34.6	8.23	101.4	204
04.02.19	8,220	25.1	34.5	8.47	101.4	210
08.02.19	8,277	25.2	34.6	8.24	100.7	205
11.02.19	8,286	24.9	34.5	8.26	100.5	205
15.02.19	8,177	23.8	34.5	8.78	102.2	215
18.02.19	8,185	24.4	34.5	8.43	100.1	207
21.02.19	8,362	24.1	34.4	8.72	103	213
25.02.19	8,215	24.2	34.5	8.70	101.4	214
01.03.19	8,322	24.5	34.5	8.51	102.5	211
04.03.19	8,323	24.4	34.5	8.23	101.8	203
06.03.19	8,370	24.4	34.5	8.57	104.2	211

ET 12

Date	pH	T	Salinity	O2		
[dd.mm.yyyy]		[°C]		[mg/l]	[%]	[mbar]
26.10.18	8,233	24.5	34.8	8.32	102.1	207
29.10.18	8,224	24.9	34.7	8.40	101.7	209
02.11.18	8,204	25.1	34.8	8.35	101.3	208
05.11.18	8,205	25.1	34.7	8.41	102.1	209
09.11.18	8,219	24.9	35.0	8.40	102.1	210
12.11.18	8,212	25.0	34.7	8.42	102.4	210
16.11.18	8,163	25.1	34.8	8.54	101.7	213
19.11.18	8,198	24.9	34.9	8.47	101.3	210
23.11.18	8,251	27.1	35.2	8.09	101.8	209
26.11.18	8,205	22.3	34.6	8.91	102.6	212
30.11.18	8,176	24.4	34.5	8.51	101.6	209
03.12.18	8,209	24.3	34.7	8.41	101.7	206
07.12.18	8,310	24.4	34.0	8.38	101.7	207
10.12.18	8,318	24.2	34.0	8.68	101.4	209
14.12.18	8,311	24.4	34.0	8.61	102.0	212
17.12.18	8,298	24.2	34.1	8.52	101.3	209
21.12.18	8,324	24.5	34.1	8.32	102.0	206
27.12.18	8,286	24.8	34.1	8.59	102.6	213
07.01.19	8,285	24.8	34.5	8.57	101.8	213
11.01.19	8,252	24.7	34.4	8.50	101.7	210
14.01.19	8,323	24.8	34.6	8.50	102.6	210
18.01.19	8,306	25.2	34.4	8.39	101.3	209
21.01.19	8,289	24.9	34.4	8.53	102.4	212
25.01.19	8,235	24.3	34.5	8.38	100.6	207
28.01.19	8,223	25.0	34.4	8.14	101.5	203
01.02.19	8,251	24.8	34.4	8.24	101.6	204
04.02.19	8,239	25.1	34.4	8.43	100.7	209
08.02.19	8,288	25.3	34.4	8.21	100.2	205
11.02.19	8,301	25.0	34.4	8.23	100.3	204
15.02.19	8,200	24.2	34.4	8.72	102.4	215
18.02.19	8,211	24.8	34.4	8.33	99.7	207
21.02.19	8,374	24.1	34.3	8.84	104.2	217
25.02.19	8,242	23.2	34.4	8.83	101.0	214
01.03.19	8,329	24.7	34.3	8.55	103.0	212
04.03.19	8,334	24.5	34.5	8.24	101.7	203
06.03.19	8,371	24.5	34.4	8.43	103.3	209

Supplementary Table S3. Experimental tank parameters.

Microplastic experimental tank measurements of pH (NBS Scale) and Salinity (S_P) before and after exposure. Temperature was kept constant at 25 °C. ET09 = Exposure 1 (E1), ET10 = Exposure 2 (E2), ET11 = Control 3 (C3), ET12 = Control 4 (C4)

Date	Tank	pH	Salinity
23.10.2018	C3	8.1	34.9
	C4	8.14	34.9
	E1	8,174	34.5
	E2	8,168	34.3
24.10.2018	C3	8,210	35.3
	C4	8,238	35.7
	E1	8,244	35.6
	E2	8,245	35.8
06.11.2018	C3	8,134	34.7
	C4	8,141	34.9
	E1	8,139	34.8
	E2	8,151	34.8
07.11.2018	C3	8,128	35.0
	C4	8,151	35.2
	E1	8,163	35.2
	E2	8,185	35.3
22.11.2018	C3	8,171	34.1
	C4	8,172	34.6
	E1	8,222	34.4
	E2	8,214	34.6
23.11.2018	C3	8,176	34.4
	C4	8,199	35.0
	E1	8,201	35.0
	E2	8,211	35.2
04.12.2018	C3	8.144	34,8
	C4	8,165	34,8
	E1	8,157	34,8
	E2	8,148	34,8
05.12.2018	C3	8,140	35,3
	C4	8,167	35,3
	E1	8,193	35,4
	E2	8,136	35,3
18.12.2018	C3	8,234	33,8
	C4	8,243	34,0
	E1	8,239	34,7
	E2	8,247	34,4
19.12.2018	C3	8,266	34,2

	C4	8,266	34,5
	E1	8,304	35,2
	E2	8,295	35,0
03.01.2019	C3	8,273	34,8
	C4	8,293	34,8
	E1	8,290	34,8
	E2	8,296	34,8
04.01.2019	C3	8,249	34,9
	C4	8,288	35,2
	E1	8,303	35,4
	E2	8,307	35,4
15.01.2019	C3	8,281	34,2
	C4	8,282	34,4
	E1	8,292	33,9
	E2	8,294	34,5
16.01.2019	C3	8,286	34,5
	C4	8,287	34,8
	E1	8,311	34,4
	E2	8,325	35,0
31.01.2019	C3	8,180	34,4
	C4	8,201	33,9
	E1	8,175	34,1
	E2	8,194	34,4
01.02.2019	C3	8,159	34,9
	C4	8,184	34,4
	E1	8,200	34,7
	E2	8,232	35,0
12.02.2019	C3	8,160	34,6
	C4	8,152	34,7
	E1	8,149	34,6
	E2	8,163	34,8
13.02.2019	C3	8,231	35,1
	C4	8,224	35,2
	E1	8,203	35,2
	E2	8,236	35,5
26.02.2019	C3	8,250	34,7
	C4	8,238	34,8
	E1	8,282	34,7
	E2	8,346	34,8
27.02.2019	C3	8,323	35,1
	C4	8,315	34,8
	E1	8,330	35,2
	E2	8,331	35,3
